# Ravens, New Caledonian crows and jackdaws parallel great apes in motor self-regulation despite smaller brains

**DOI:** 10.1098/rsos.160104

**Published:** 2016-04-20

**Authors:** Can Kabadayi, Lucy A. Taylor, Auguste M. P. von Bayern, Mathias Osvath

**Affiliations:** 1 Department of Cognitive Science, Lund University, Helgonavägen 3, 221 00 Lund, Sweden; 2 Department of Zoology, University of Oxford, South Parks Road, Oxford, UK; 3 Max Planck Institute for Ornithology, 82319 Seewiesen, Germany

**Keywords:** inhibition, motor self-regulation, corvid cognition, self-control, avian brains, *Corvus*

## Abstract

Overriding motor impulses instigated by salient perceptual stimuli represent a fundamental inhibitory skill. Such motor self-regulation facilitates more rational behaviour, as it brings economy into the bodily interaction with the physical and social world. It also underlies certain complex cognitive processes including decision making. Recently, MacLean *et al*. (MacLean *et al.* 2014 *Proc. Natl Acad. Sci. USA* 111, 2140–2148. (doi:10.1073/pnas.1323533111)) conducted a large-scale study involving 36 species, comparing motor self-regulation across taxa. They concluded that absolute brain size predicts level of performance. The great apes were most successful. Only a few of the species tested were birds. Given birds' small brain size—in absolute terms—yet flexible behaviour, their motor self-regulation calls for closer study. Corvids exhibit some of the largest relative avian brain sizes—although small in absolute measure—as well as the most flexible cognition in the animal kingdom. We therefore tested ravens, New Caledonian crows and jackdaws in the so-called *cylinder task*. We found performance indistinguishable from that of great apes despite the much smaller brains. We found both absolute and relative brain volume to be a reliable predictor of performance within Aves. The complex cognition of corvids is often likened to that of great apes; our results show further that they share similar fundamental cognitive mechanisms.

## 1. Introduction

*Executive functions* refer to a cluster of top-down cognitive expressions often contrasted with automatized, instinctual and conditioned processes [1]. These functions require cognitive effort: not only do they inhibit prepotent responses, but also they sometimes guide choice among several options. Some are cognitively more taxing than others. Self-control—commonly defined as the ability to decline an immediate small reward in favour of a larger future one—ranks among the most demanding. It requires deciding among options of differing values in relation to a temporal dimension. At the other end of the taxation spectrum, one finds, e.g. motor self-regulation, which requires only the hindering of a movement: one that is not immediately rewarding but does not infer major cost [2].

Self-control is essential to such complex cognitive skills as decision-making and planning [3,4]. Without that competence, an animal becomes—in Roberts' [5] words—*stuck in time*. Currently, those groups of non-human animals that stand out for their levels of self-control are great apes, corvids and parrots [6–8].

Because self-control is an underlying cognitive process enabling spatio-temporal detachment across various contexts, its evolution and expression in different taxa inform a broader view on cognitive evolution. Self-control is underpinned by—among other things—motor self-regulation. Without well-developed regulation, self-control will not come about easily. Studying such basic inhibition comparatively across closely and distantly related taxa has its merits. It helps establish whether there is a link between levels of motor self-regulation and levels of self-control, and other cognitive skills.

MacLean and co-workers [9] compared motor self-regulatory tasks for 36 species, mostly primates. The tasks were designed to make certain motor responses tempting to execute but counterproductive to the goal. Although the authors refer to these tasks as tests of self-control, this does not conform to definitions found in the literature, as the tasks did not involve decisions between two options, one resulting in an immediate small reward and another in a larger but delayed one. We agree with Beran [2] that what they actually tested was motor self-regulation, as the tasks required only the inhibition of prepotent responses. Performance levels were investigated for correlation with absolute versus relative brain size, dietary breadth and social complexity. Great apes showed the highest levels of inhibition. Across all species, absolute brain size was the best predictor of success; brain size relative to body mass had no significant effect. That said, relative brain size did predict performance among the primates, though not as strongly as absolute size.

Of the 36 species tested, 29 were mammals out of which 23 were primates. Only seven were birds. The authors cautioned that, as the findings were primate dominated, they might not generalize. We therefore believe that testing more bird species would be helpful in at least two ways. Firstly, to establish whether the relationship between absolute brain size and inhibition performance holds across taxa (i.e. whether birds perform more poorly due to smaller absolute brain size) and, secondly, whether a similar pattern emerges between brain size and motor self-regulation within Aves.

MacLean and co-workers [9] included two species from corvid genera—Eurasian jays (*Garrulus glandarius*) and western scrub jays (*Aphelocoma californica*)—but no members of the genus *Corvus*. We tested three species of crows belonging to the genus *Corvus*: ravens (*Corvus corax*), New Caledonian crows (*Corvus moneduloides*) and jackdaws (*Corvus monedula*).

Including more corvids is informative for several reasons. Corvids have one of the largest relative brain sizes among birds [10], while ravens have the largest absolute brain size among corvids: roughly three times that of the jackdaw and twice that of the New Caledonian crow. Corvids have often been likened to the great apes in terms of complex cognition involved in physical, social and memory skills [11] as well as self-control tasks [7]. By comparing primate and corvid data, one can approach the question whether the complex cognition seen in these phylogenetically distant taxa can be explained in part by similar underlying proximate mechanisms, such as basic motor self-regulatory skills [12].

MacLean and co-workers used two tasks: the cylinder task and the *A*-not-*B* task. In the cylinder task, subjects are familiarized with an opaque hollow cylinder with openings at both ends and a reward in the middle. Once subjects learn to take the reward through the openings, a transparent cylinder replaces the opaque one. A direct reach for the visible reward—as opposed to detouring to one of the openings—counts as inhibition failure. The task was originally developed to study ontogeny of inhibition skills in humans and rhesus monkeys [13] and later adopted for other animals [14].

Piaget designed the *A*-not-*B* task [15] to study infants' developing concept of object permanence. It was subsequently adapted to investigate a wide range of skills including means--end reasoning, short-term memory use and inhibitory control [16,17]. Subjects get repeated experience retrieving a reward placed under opaque container *A*. In test trials, the experimenter moves the reward from *A* to container *B* in full view of the subject, and then lets it search for the reward. The choice of *A* instead of *B* marks an error. Some regard this as an inhibition failure built on preceding habit: the habit of retrieving the reward from *A* must be inhibited when the reward is moved to *B*.

We did not use the *A*-not-*B* task for two main reasons: it is not clearly a motor self-regulatory task, and MacLean *et al*'s study had methodological shortcomings regarding its use. Solving the task is an exceedingly dynamic process affected by such contextual features as the delay between hide and search, the visual distinctiveness—as well as number—of containers, and the level of attention directed towards the baited container [18,19]. Given this dynamic complexity, the *A*-not*-B* task has been widely questioned as an inhibitory task [18]. In the same vein, Jelbert and colleagues recently showed that New Caledonian crows who received training in tracking human hand actions were significantly better at solving the *A-*not*-B* task than those who were not trained [20].

MacLean *et al*. also implemented the task unsatisfactorily. Firstly, they conducted only a single trial per individual, compared to 10 trials per individual in the cylinder task. Inhibitory control is not a binary skill but a continuum—as shown in the cylinder task. Secondly, they used three containers, of which the middle container was never rewarded. As we understand it, every failure to retrieve the reward—including searching the middle container—was regarded as inhibitory failure. The middle location should not be regarded as an inhibition failure as no habit was formed in relation to it. Thirdly, as many as nine of the species examined could not be tested in the *A*-not-*B* task. Only four of the seven bird species received the A-not-B task. Overall the task does not provide much informative data.

We therefore replicated only the cylinder task conducted by MacLean and co-workers [9], comparing the performance on it in the same species, with particular focus on the birds. It must be noted, however, that MacLean and co-workers’ findings regarding the correlation between absolute and relative brain size holds for this task alone. In our replication, we followed the experimental procedures described in MacLean *et al*.

## 2. Material and methods

### 2.1. Subjects and housing

Five adult ravens (three females), 10 adult New Caledonian crows (four females) and 10 adult jackdaws (five females) participated in the study. The ravens were tested at Lund University Corvid Cognition Station in Sweden, the other birds at the Avian Cognition Research Station associated with the Max Planck Institute for Ornithology, Seewiesen, Germany. All were housed in environmentally enriched outdoor aviaries. The New Caledonian crows had access to heated indoor compartments (6 m^2^). The ravens and jackdaws were housed as a social group in 400 m^2^ (ravens) and 120 m^2^ (jackdaws) spaces, and the New Caledonian crows as pairs in 18–32 m^2^ spaces. All birds had ad libitum access to food and water. Subjects were tested individually in familiar testing compartments.

### 2.2. Set-up and materials

The cylinder task employed two apparatuses: an opaque and a transparent hollow cylinder. The cylinders were open on both sides and attached to a wooden base. The openings allowed the subjects to insert their heads and retrieve a highly desirable food reward from the middle of the cylinder.

### 2.3. Procedure

First the birds were habituated to the opaque cylinder by having it placed inside the aviary. A bird was defined as habituated when it interacted with the cylinder. All subjects except two jackdaws and one raven habituated after 2 days. It took 3 days for two jackdaws and 6 days for one raven to habituate. Familiarization trials ensued 1 day after the habituation. In these trials, the birds were familiarized with the opaque cylinder and its particular shape. The experimenter hid the reward at the centre of the tube while the subject observed; then the subject was allowed to retrieve the reward. Correct responses were coded, for the first responses, as reaching through either opening to get the reward. Regardless of their first response, subjects were eventually allowed to obtain the reward. To proceed to the test trials, subjects had to succeed on four out of five consecutive trials. All birds completed the familiarization trials within one session in 1 day and they did not have any incorrect responses. Testing trials followed 1 day after the familiarization trials.

In the test trials, a transparent cylinder replaced the opaque cylinder. Ten trials were administered for every subject. The baiting procedure was identical to that used in the familiarization trials. Correct responses were coded, again for the first responses, when one of the openings was used to retrieve the reward (detour through the side), and an incorrect response was coded if physical contact was made with the front of the cylinder (probably in an attempt to directly reach for the reward). Subjects were eventually allowed to retrieve the reward regardless of the accuracy of their first responses.

## 3. Results

All ravens succeeded on all trials (100%). The jackdaws averaged 97% success (min = 8, max = 10), the New Caledonian crows 92% (min = 7, max = 10). The results are comparable with those of the great apes: chimpanzees (100%), orangutans (99.1%), bonobos (95%) and gorillas (94.4%) (table 1).

**Table 1.** The left table shows the top 10 performing species in the cylinder task in MacLean *et al*'s study [9]. The right table shows new top 10 ranking when including the performance of the *Corvus* species in the current study (shown in bold).

rank	species	cylinder task: average score	endocranial cm^3^ volume	rank	species	cylinder task: average score	endocranial cm^3^ volume
ranking MacLean *et al.* [9]				new ranking including *Corvus* species			
1	chimpanzee	100.0	368.4	1	chimpanzee	100.0	368.4
2	orangutan	99.1	377.4	**=1**	**raven**	**100**.**0**	**14**.**5**
3	capuchin monkey	95.9	66.6	3	orangutan	99.1	377.4
4	bonobo	95.0	341.3	**4**	**jackdaw**	**97**.**0**	**5**.**2**
=4	coyote	95.0	85.2	5	capuchin monkey	95.9	66.6
6	gorilla	94.4	490.4	6	bonobo	95.0	341.3
7	rhesus macaque	80.0	89.0	=6	coyote	95.0	85.2
8	domestic dog	79.1	87.0	8	gorilla	94.4	490.4
9	gray wolf	77.3	127.1	**9**	**New Caledonian crow**	**92**.**0**	**7**.**3**
10	western scrub jay	76.7	2.9	10	rhesus macaque	80.0	89.0

To perform detailed comparison, we obtained unpublished trial-level results on the seven bird species from MacLean *et al*., along with endocranial brain volume and body mass data from the literature concerning the three *Corvus* species [21–23] analysed together with the mass and volume data for the other seven bird species reported in the replicated study (MacLean *et al*. supporting information table S1). Two generalized linear mixed-effect models (GLMM) were constructed to investigate the effect of absolute and residual brain volume on performance, with the latter calculated as the residual from a phylogenetic regression of absolute-brain-volume predicted body mass. For both models, trial number was added as a fixed effect. Both absolute and residual brain volume as well as trial number were found to be significant predictors of performance on the cylinder task (table 2). Comparison of the two models using the Akaike information criterion (AIC) provided stronger support for the absolute brain volume model (table 2). In addition, we investigated the effect of the trial number on performance separately for all bird species tested. We found a trial effect on performance in four of the bird species examined by MacLean *et al*., with no such effect in the *Corvus* species we examined. Performance improved significantly over successive trials for the zebra finches (*p* = 0.037), song sparrows (*p* = 0.014), swamp sparrows (*p* = 0.008) and orange amazons (*p* = 0.029; electronic supplementary material, table S1).

**Table 2.** Summary of the two final models that investigated the effect of absolute brain volume, residual brain volume and trial number on the cylinder task performance for 10 bird species tested in this study and MacLean *et al*. [9].

predictor	effect ± s.e.	*p*-value	χ^2^	d.f.	AIC
absolute brain volume (log)	0.664 ± 0.107	<0.001	57.01	2	1808.22
trial	0.109 ± 0.022	<0.001			
residual brain volume (log)	1.115 ± 0.413	0.007	27.94	2	1837.28
trial	0.107 ± 0.022	<0.001			

## 4. Discussion

**Figure 1.**
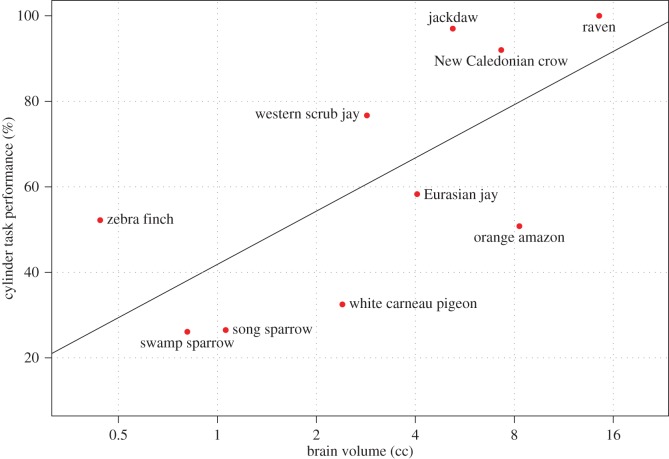
The relationship between the absolute brain size and the cylinder task performance for 10 bird species tested in this study and MacLean *et al*. [9]. The trend line is based on a species mean percentage score on the cylinder task.

The *Corvus* species performed on a similar level to the great apes, despite vastly smaller absolute brain sizes. A chimpanzee brain is roughly 26 times larger than a raven's; nevertheless, both species achieve 100% success. The jackdaws were more successful than either the bonobos or gorillas, despite a brain 70–94 times smaller. Clearly, absolute brain size is no overall predictor of motor self-regulation across a wider range of animal taxa. However, as among primates, absolute brain size does appear to be a significant predictor across bird species (figure 1), but on the other hand relative brain size is as well a significant predictor within birds.

Recent findings indicate the existence of different scaling relationships between total number of neurons and absolute brain size across mammalian taxa [24]: bigger brains do not necessarily contain more neurons than smaller ones. Neuronal numbers might perhaps better explain corvids' and apes' similar performance in the cylinder task than absolute or relative brain size, but such investigations have to await studies on neuronal counts in bird brains. Furthermore, the crude comparisons made both in our study and MacLean *et al*.'s may miss the importance of the relative or absolute sizes of certain brain regions related to inhibitory skills. The lack of data on different brain regions across bird species meant that we could not perform the requisite analysis.

Regardless of any comparisons of various brain measurements, one can conclude that the similarities between corvids and great apes in complex cognitive skills [11] also hold for at least one less taxing skill: motor self-regulation.

Some of the bird results from the cylinder task in MacLean *et al*.'s study are difficult to interpret. For example, we found a significant positive trial effect for four of the bird species; see the electronic supplementary material for details. This might indicate that the subjects representing these species lacked sufficient initial experience of the task but gained experience across trials. The cylinder task is not suitable if adequate experience is lacking: i.e. failures might result from other causes than limited inhibition. It has also been shown in human children that previous experience with transparent objects is crucial for this task [19]; therefore, one should not administer the cylinder task if it is not clearly established that the subject has sufficient experience about transparent surfaces. Recently, it was shown that Clark's nutcrackers (*Nucifraga columbiana*) perform better in the last five, out of the 10, trials in the cylinder task, even if they have passed the familiarization trials with an opaque cylinder; the authors concluded that this was an effect of learning about transparent surfaces [25]. We do not know whether similar trial effects hold for some of the mammalian species. Here it is worth mentioning that all our birds have had previous experience of transparent surfaces, both from various enrichment activities and experiments since they were young.

One might reasonably ask not only for reports on failures and successes, but also for information on the behavioural dynamics of how the tasks were solved by different species. Not all of the few failures reported from the *Corvus* species in our study were ‘clean’ failures such that the bird tried to reach the reward directly as if the barrier was air. Instead, some birds nudged the cylinder, while others behaved as if exploring the attachment of the cylinder. Without details of possible exploratory behaviour across species, it might be premature to make the far-reaching conclusions about evolution and brain size that MacLean *et al*. do [26]. What is without doubt is that great apes and *Corvus* corvids have pronounced motor self-regulatory behaviour in relation to the cylinder task, despite very different absolute different brain sizes.

## Supplementary Material

Title: Supplement on the description of statistical analyses and the supporting data The supplementary material provides detailed information on the statistical analyses as well as the complete dataset in which the statistical analyses were conducted. It also contains information about the material used in the study as well as a regression output table documenting the trial effect for 9 species.

## Data accessibility

The datasets supporting this article have been uploaded as part of the electronic supplementary material.
