# Supplementary material for: Ravens, New Caledonian crows and jackdaws parallel great apes in motor self-regulation despite smaller brains
Source: R Soc Open Sci. 2016 Apr 20;3(4):160104. doi: 10.1098/rsos.160104 (PMC4852647; doi:10.1098/rsos.160104)
Supplement: Title: Supplement on the description of statistical analyses and the supporting data The supplementary material provides detailed information on the statistical analyses as well as the complete dataset in which the statistical analyses were conducted. It also contains information about the material  [file rsos160104supp1.pdf]

## Supplementary Material

*Ravens, New Caledonian crows and jackdaws parallel great apes in motor self-regulation despite smaller brains*

Can Kabadayi, Lucy A. Taylor, Auguste M.P. von Bayern and Mathias Osvath

## Subjects

Five ravens (three females, two males), ten jackdaws (five females, five males) and ten New Caledonian crows (four females, six males) took part in the study. All birds were adults.

## Material and Methods

Two types of hollow cylinders were used: one opaque and one transparent (Fig. S1). The cylinders had openings on both ends and were attached to a wooden base so that the subjects had to move to the side of them to reach through to the reward placed in the center. The diameter of the cylinders were large enough to allow the subjects to enter head first and retrieve the reward (5.5 cm for jackdaws and New Caledonian crows, 9 cm for ravens) and they were long enough so that subjects had to insert most of the head inside the cylinders to retrieve the reward (12 cm for the New Caledonian crows and ravens, 10 cm for jackdaws).

Testing trials were administered within a single day in two 5-trial sessions (one in the morning and one in the afternoon). Subjects were given maximum 3 minutes to make a choice after the cylinder was baited. For the vast majority of the trials subjects immediately approached and interacted with the cylinder once it was baited. One New Caledonian and one jackdaw trial were stopped as the birds failed to interact with the cylinder within 3 minutes. Two jackdaw and two raven trials were stopped due to environmental disturbances. In those cases the trials were stopped they continued in the next session. All jackdaws and all New Caledonian crows finished the testing trials within 24 hours. One raven had four days between the two sessions. Another raven completed the testing phase in three sessions (5, 3 and 2 trials per session respectively, with four days between session 1 and session 2 and three days in between the last session).

## Statistical analysis

We performed a phylogenetic generalized least squares (PGLS) regression to predict brain size from body mass. This type of regression controls for species' non-independence due to phylogenetic relatedness. For PGLS we specified a correlation structure derived from the phylogenetic tree for ten bird species based on http://birdtree.org/subsets. The residuals from the analysis were used as a measure of residual brain volume.

The replicated study only reported species' average score for the cylinder task and not individual performances over the ten trials, which might be problematic given the vast differences in number of subjects tested per species. To conduct more detailed analysis within bird species allowing us to control for variance and trial effect, we gratefully obtained individual trial data for the seven bird species tested from the following authors on that paper: Ljerka Ostojic (Eurasian jay and Western scrub jay), Rindy Anderson (zebra finch, song sparrow and swamp sparrow), Thomas Zentall (white carneau pigeon), and Amanda Seed (orange-winged amazon) (Table S3).

We then combined this data with that which we obtained from the three *Corvus* species (Table S2) and performed regression analysis where the dependent variable was success (1) or failure (0) on a cylinder task trial. Two generalized linear mixed-effect models (GLMM) fitted for binary

data were constructed to investigate the effect of absolute and residual brain volume on cylinder-task performance. For both models, we included trial number and brain-size measures as well as their interactions as fixed-effects. We used the method of stepwise elimination of the non-significant effects using log-likelihood ratio tests to obtain the final model. For both models, the interaction of the two fixed effects was not significant and therefore excluded. The two remaining fixed effects were significant for both models. In order to decide which of the main effects better explained the cylinder task performance we used Akaike information criterion (AIC). Lower AIC values indicate an increased support for the model. For the regression analysis, absolute and residual brain volumes were log-transformed.

We investigated the trial effect on cylinder-task performance separately for all species – except ravens, as their performance did not vary over the trials (Table S1). This analysis was conducted by constructing GLMM fitted for binary data for all nine species with the cylinder task performance as the dependent variable and trial number as fixed-effect. Since the data included repeated measures for individual subjects, "individual" was added as a random effect to all models.

All statistical analysis was conducted in *R* (v.3.0.3, the R Foundation for Statistical Computing: http://www.R-project.org) using the packages lme4, nlme and APE. Significance level was set at 0.05.

## Tables and Figures

**Figure S1:** Cylinder types used in the task: opaque (a) and transparent (b).

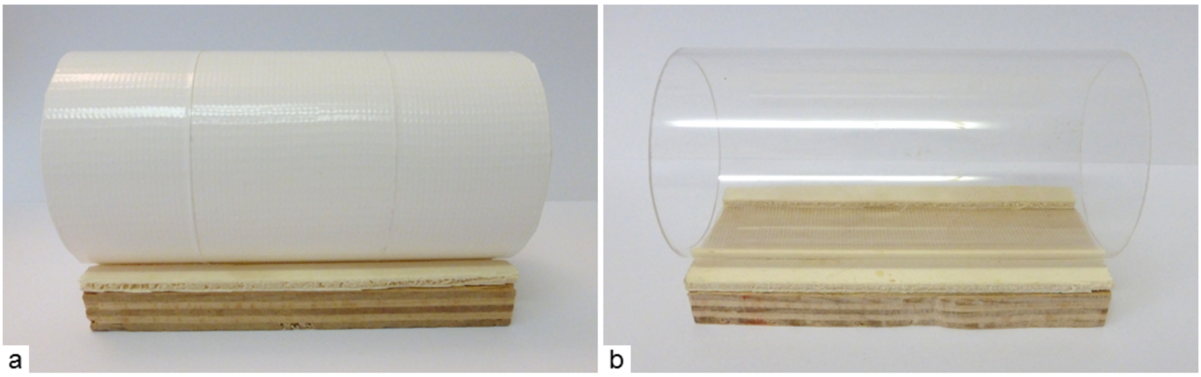

**Table S1:** Output of the GLMM analysis documenting the effect of the trial number on the cylinder task performance analyzed separately for 9 different bird species. Ravens were excluded from the analysis because there was no variation in their performance over trials.

| Species | Number of individuals | Effect | SE | z | p |
|---|---|---|---|---|---|
| Jackdaw | 10 | - 1.028 | 2.089 | - 0.492 | 0.623 |
| New Caledonian crow | 10 | - 0.212 | 0.261 | - 0.815 | 0.414 |
| Song sparrow | 20 | 0.167 | 0.068 | 2.465 | 0.014* |
| Swamp sparrow | 23 | 0.024 | 0.088 | 2.660 | 0.008* |
| Eurasian jay | 6 | 0.059 | 0.145 | 0.410 | 0.682 |
| Western scrub-jay | 6 | 0.190 | 0.122 | 1.568 | 0.117 |
| Orange amazon | 12 | 0.228 | 0.104 | 2.183 | 0.029* |
| White carneau pigeon | 11 | 0.155 | 0.122 | 1.272 | 0.204 |
| Zebra finch | 47 | 0.068 | 0.033 | 2.089 | 0.037* |

**Table S1**.

**Table S2:** Individual information and summary of the cylinder task performance for three *Corvus* species tested in our study.

| Species | Individual | Sex | Age (years) | Performance out of 10 | Trial no of the error |
|---|---|---|---|---|---|
| Raven | Siden | male | 3 | 10 | |
| Raven | None | female | 3 | 10 | |
| Raven | Rikard | male | 3 | 10 | |
| Raven | Juno | female | 3 | 10 | |
| Raven | Tosta | female | 2 | 10 | |
| Jackdaw | Chapa | female | 3 | 8 | 7,10 |
| Jackdaw | Mohawk | male | 3 | 9 | 10 |
| Jackdaw | Chocktaw | female | 3 | 10 | |
| Jackdaw | Collins | male | 7 | 10 | |
| Jackdaw | Jackomo | female | 11 | 10 | |
| Jackdaw | Apache | female | 3 | 10 | |
| Jackdaw | Pronto | female | 7 | 10 | |
| Jackdaw | Blackfoot | male | 3 | 10 | |
| Jackdaw | Chock | male | 10 | 10 | |
| Jackdaw | Cherokee | male | 3 | 10 | |
| New Caledonian crow | Admiral | male | wild-caught adult | 8 | 5,10 |
| New Caledonian crow | Agaios | male | wild-caught adult | 9 | 9 |
| New Caledonian crow | Tabou | female | 5 | 10 | |
| New Caledonian crow | Tumulte | female | 5 | 10 | |
| New Caledonian crow | Jungle | male | 5 | 10 | |
| New Caledonian crow | Mangroove | male | wild-caught adult | 7 | 4,6,8 |
| New Caledonian crow | Papaye | male | wild-caught adult | 9 | 1 |
| New Caledonian crow | Liane | female | 5 | 10 | |
| New Caledonian crow | Tortue | female | wild-caught adult | 9 | 10 |
| New Caledonian crow | Mango | male | wild-caught adult | 10 | |

**Table S3:** Summary of the cylinder task performance for seven bird species provided by the authors mentioned above.

| Species | Individual | Performance out of 10 | Trial no of the error |
|---|---|---|---|
| Eurasian jay | Quito | 6 | 1,3,4,7 |
| Eurasian jay | Ohorougu | 4 | 3,5,6,8,9,10 |
| Eurasian jay | Wilson | 2 | 1,2,4,5,6,7,9,10 |
| Eurasian jay | Hoy | 10 | |
| Eurasian jay | Hunter | 6 | 4,5,6,10 |
| Eurasian jay | Romero | 7 | 1,3,6 |
| Western scrub-jay | 223 | 7 | 1,2,8 |
| Western scrub-jay | 224 | 8 | 6,8 |
| Western scrub-jay | 222 | 10 | |
| Western scrub-jay | 201 | 6 | 2,3,4,8 |
| Western scrub-jay | 203 | 6 | 1,2,7,8 |
| Western scrub-jay | 229 | 9 | 1 |
| Orange winged amazon | Benny | 4 | 1,2,3,4,6,10 |
| Orange winged amazon | Stumpy | 5 | 1,5,7,9,10 |
| Orange winged amazon | Pete | 5 | 1,5,7,9,10 |
| Orange winged amazon | Bo | 4 | 1,2,6,7,8,9 |
| Orange winged amazon | Tulip | 4 | 1,2,3,6,7,8 |
| Orange winged amazon | Ricky | 5 | 1,2,3,5,6 |
| Orange winged amazon | Simon | 4 | 1,2,3,4,5,10 |
| Orange winged amazon | Piglet | 7 | 2,6,7 |
| Orange winged amazon | Penny | 7 | 2,7,10 |
| Orange winged amazon | Belle | 5 | 1,2,3,6,8 |
| Orange winged amazon | Rocky | 4 | 1,2,3,4,5,6 |
| Orange winged amazon | Freckles | 7 | 1,5,6 |
| White carneau pigeon | 10742 | 0 | 1,2,3,4,5,6,7,8,9,10 |
| White carneau pigeon | 22642 | 2 | 1,2,4,5,6,7,8,10 |
| White carneau pigeon | 17878 | 2 | 1,2,3,4,6,7,9,10 |
| White carneau pigeon | 19306 | 2 | 1,3,4,5,6,7,8,10 |
| White carneau pigeon | 11746 | 7 | 6,7,9 |
| White carneau pigeon | 19227 | 6 | 1,3,7,8 |
| White carneau pigeon | 19845 | 6 | 1,2,3,4 |
| White carneau pigeon | 19824 | 7 | 1,2,3 |
| White carneau pigeon | 19276 | 0 | 1,2,3,4,5,6,7,8,9,10 |
| White carneau pigeon | 19849 | 4 | 1,2,4,5,7,9 |
| White carneau pigeon | 19338 | 2 | 1,2,3,5,6,8,9,10 |
| Swamp sparrow | Sw644 | 0 | 1,2,3,4,5,6,7,8,9,10 |
| Swamp sparrow | Sw665 | 1 | 1,2,3,4,6,7,8,9,10 |
| Swamp sparrow | Sw661 | 3 | 1,2,3,4,5,6,9 |
| Swamp sparrow | Sw662 | 1 | 1,2,3,4,5,6,7,8,9 |
| Swamp sparrow | Sw654 | 3 | 1,3,5,6,7,8,10 |
| Swamp sparrow | Sw656 | 6 | 1,2,6,7 |
| Swamp sparrow | Sw640 | 0 | 1,2,3,4,5,6,7,8,9,10 |
| Swamp sparrow | Sw645 | 4 | 1,2,6,7,9,10 |

| | | | |
|---|---|---|---|
| Swamp sparrow | Sw641 | 2 | 1,2,3,4,5,6,7,10 |
| Swamp sparrow | Sw 652 | 1 | 1,2,3,4,5,7,8,9,10 |
| Swamp sparrow | Sw 643 | 0 | 1,2,3,4,5,6,7,8,9,10 |
| Swamp sparrow | Sw 642 | 4 | 1,2,4,6,7,9 |
| Swamp sparrow | Sw 655 | 6 | 1,3,4,9 |
| Swamp sparrow | Sw 650 | 3 | 1,4,5,6,8,9,10 |
| Swamp sparrow | Sw 646 | 2 | 2,3,4,5,6,8,9,10 |
| Swamp sparrow | Sw 657 | 0 | 1,2,3,4,5,6,7,8,9,10 |
| Swamp sparrow | Sw 663 | 3 | 1,2,3,4,5,7,8 |
| Swamp sparrow | Sw 658 | 3 | 1,3,4,5,7,8,9 |
| Swamp sparrow | Sw 666 | 2 | 1,2,3,4,5,6,7,8 |
| Swamp sparrow | Sw 667 | 2 | 1,2,3,4,5,6,7,8 |
| Swamp sparrow | Sw 669 | 7 | 1,5,6 |
| Swamp sparrow | Sw 668 | 1 | 1,2,3,4,5,6,7,8,9 |
| Swamp sparrow | Sw 659 | 4 | 1,2,5,6,7,10 |
| Song sparrow | Bk-OR | 1 | 1,2,3,4,5,6,7,8,9 |
| Song sparrow | FY-WW | 1 | 1,2,4,5,6,7,8,9,10 |
| Song sparrow | G-BkBk | 2 | 1,2,4,5,6,7,8,9 |
| Song sparrow | G-OO | 2 | 1,2,3,4,6,8,9,10 |
| Song sparrow | G-PkPk | 2 | 2,3,4,5,6,7,8,10 |
| Song sparrow | G-WG | 5 | 1,2,3,4,7 |
| Song sparrow | O-BkBk | 7 | 2,4,9 |
| Song sparrow | O-IBIIBI | 5 | 6,7,8,9,10 |
| Song sparrow | O-PuPu | 0 | 1,2,3,4,5,6,7,8,9,10 |
| Song sparrow | Pu-BkBk | 2 | 2,3,4,5,6,7,8,10 |
| Song sparrow | Pu-RR | 3 | 1,2,3,4,5,7,9 |
| Song sparrow | R-WdBl | 4 | 1,2,6,7,8,9 |
| Song sparrow | W-OW | 0 | 1,2,3,4,5,6,7,8,9,10 |
| Song sparrow | W-PkPk | 1 | 1,2,3,4,5,6,7,9,10 |
| Song sparrow | W-PuW | 2 | 1,2,4,5,6,7,9,10 |
| Song sparrow | W-RW | 2 | 1,2,3,4,5,6,8,9 |
| Song sparrow | Y-BkBk | 6 | 1,2,8,9 |
| Song sparrow | Y-dBIdBI | 4 | 2,3,4,5,6,10 |
| Song sparrow | Y-OO | 3 | 1,3,4,5,8,9,10 |
| Song sparrow | Y-RY | 1 | 1,2,3,4,5,6,7,8,10 |
| Zebra finch | zf105 | 5 | 1,3,7,8,9 |
| Zebra finch | zf104 | 4 | 2,3,4,8,9,10 |
| Zebra finch | zf103 | 7 | 1,2,8 |
| Zebra finch | zf102 | 4 | 3,4,5,7,8,10 |
| Zebra finch | zf100 | 7 | 4,7,9 |
| Zebra finch | zf 99 | 7 | 4,5,6 |
| Zebra finch | zf 98 | 4 | 1,4,5,6,8,9 |
| Zebra finch | zf 97 | 7 | 2,3,7 |
| Zebra finch | zf 96 | 7 | 2,5,8 |
| Zebra finch | zf 94 | 3 | 1,2,4,6,7,9,10 |
| Zebra finch | zf 91 | 6 | 1,3,4,7 |
| Zebra finch | zf 89 | 7 | 2,7,10 |
| Zebra finch | zf 88 | 8 | 4,6 |

| | | | |
|---|---|---|---|
| Zebra finch | zf 87 | 6 | 3,4,6,9 |
| Zebra finch | zf 85 | 7 | 2,6,7 |
| Zebra finch | zf 83 | 5 | 1,2,4,5,9 |
| Zebra finch | zf 82 | 4 | 1,3,4,6,9,10 |
| Zebra finch | zf 81 | 3 | 1,2,5,7,8,9,10 |
| Zebra finch | zf 80 | 5 | 1,2,4,5,9 |
| Zebra finch | zf 79 | 6 | 2,3,6,10 |
| Zebra finch | zf 75 | 5 | 1,3,5,6,10 |
| Zebra finch | zf 73 | 6 | 1,2,4,7 |
| Zebra finch | zf 71 | 6 | 2,5,8,10 |
| Zebra finch | zf 70 | 5 | 3,4,5,8,9 |
| Zebra finch | zf 68 | 5 | 1,2,4,8,10 |
| Zebra finch | zf 67 | 6 | 3,4,6,8 |
| Zebra finch | zf 66 | 5 | 2,3,4,8,10 |
| Zebra finch | zf 63 | 3 | 2,3,4,5,6,7,10 |
| Zebra finch | zf 62 | 5 | 3,4,5,6,8 |
| Zebra finch | zf 61 | 4 | 1,2,3,4,7,8 |
| Zebra finch | zf 58 | 4 | 1,3,4,5,7,10 |
| Zebra finch | zf 56 | 5 | 2,3,4,6,9 |
| Zebra finch | zf 55 | 4 | 3,4,6,7,8,10 |
| Zebra finch | zf 54 | 7 | 1,2,8 |
| Zebra finch | zf 53 | 6 | 3,5,7,9 |
| Zebra finch | zf 52 | 5 | 2,3,5,6,7 |
| Zebra finch | zf 51 | 8 | 1,7 |
| Zebra finch | zf 50 | 6 | 1,2,3,9 |
| Zebra finch | zf 49 | 5 | 1,2,4,6,8 |
| Zebra finch | zf 47 | 7 | 4,5,7 |
| Zebra finch | zf 45 | 4 | 1,3,4,5,7,9 |
| Zebra finch | zf 44 | 6 | 2,3,4,10 |
| Zebra finch | zf 43 | 5 | 3,4,5,6,8 |
| Zebra finch | zf 42 | 5 | 3,6,7,8,9 |
| Zebra finch | zf 40 | 6 | 2,3,7,8 |
| Zebra finch | zf 38 | 5 | 2,3,6,8,9 |
| Zebra finch | zf 35 | 5 | 1,5,6,8,10 |
